# Endocarditis in Patients with Aortic Valve Prosthesis: Comparison between Surgical and Transcatheter Prosthesis

**DOI:** 10.3390/antibiotics10010050

**Published:** 2021-01-06

**Authors:** Micaela De Palo, Pietro Scicchitano, Pietro Giorgio Malvindi, Domenico Paparella

**Affiliations:** 1 Section of Cardiac Surgery, A.O.U. Consorziale Policlinico di Bari, 70124 Bari, Italy; 2 Section of Cardiology, F. Perinei Hospital, Altamura, 70022 Bari, Italy; piero.sc@hotmail.it; 3 Wessex Cardiothoracic Centre, University Hospital Southampton, Southampton SO16 6YD, UK; pg.malvindi@hotmail.com; 4 Division of Cardiac Surgery, Santa Maria Hospital, GVM Care & Research, 70124 Bari, Italy; 5 Department of Medical and Surgical Science, University of Foggia, 71122 Foggia, Italy

**Keywords:** endocarditis, aortic valve, TAVR, SAVR

## Abstract

The interventional treatment of aortic stenosis is currently based on transcatheter aortic valve implantation/replacement (TAVI/TAVR) and surgical aortic valve replacement (SAVR). Prosthetic valve infective endocarditis (PVE) is the most worrisome complication after valve replacement, as it still carries high mortality and morbidity rate. Studies have not highlighted the differences in the occurrence of PVE in SAVR as opposed to TAVR, but the reported incidence rates are widely uneven. Literature portrays different microbiological profiles for SAVR and TAVR PVE: Staphylococcus, Enterococcus, and Streptococcus are the pathogens that are more frequently involved with differences regarding the timing from the date of the intervention. Imaging by means of transoesophageal echocardiography, and computed tomography (CT) Scan is essential in identifying vegetations, prosthesis dysfunction, dehiscence, periannular abscess, or aorto-ventricular discontinuity. In most cases, conservative medical treatment is not able to prevent fatal events and surgery represents the only viable option. The primary objectives of surgical treatment are radical debridement and the removal of infected tissues, the reconstruction of cardiac and aortic morphology, and the restoration of the aortic valve function. Different surgical options are discussed. Fast diagnosis, the adequacy of antibiotics treatment, and prompt interventions are essential in preventing the negative consequences of infective endocarditis (IE).

## 1. Introduction

Interventional treatment of aortic stenosis is currently based on two different approaches: transcatheter aortic valve implantation/replacement (TAVI/TAVR) and surgical aortic valve replacement (SAVR) [1].

The results from randomized controlled trials (RCTs) and observational/retrospective studies pointed out the evolution of bioprostheses that are used in TAVR, as compared to SAVR, in patients with low- to moderate-high risk for surgical intervention and the need for carefully opting between the two procedures [2,3].

Furthermore, the use of biological prosthetic valves in first place should induce physicians to carefully think about the right indications for adopting TAVR, rather than SAVR. In fact, a recent meta-analysis showed that patients that were treated with bioprosthetic valves demonstrated a 60% higher risk for infective endocarditis as compared to those who underwent cardiac valve replacement treatment with mechanical prostheses [4].

Infective endocarditis (IE) is the most worrisome complication after valve replacement, as it still carries high mortality and morbidity, despite the general improvement in diagnosis, medical, and surgical treatment [5].

A meta-analysis from Abegaz et al. reported a mortality rate that ranged from 20 to 37% at short- and up to five-year follow-up, while the rate of complications due to septic embolisms, cardiac, and/or renal involvement ranged between 19 and 39% [6]. Furthermore, about 25% of patients already treated for IE might be re-hospitalized, due to recurrent cardiac valve infection [7].

Comparisons between SAVR and TAVR in terms of IE incidence and outcomes are still under investigation [3,8]. However, initial evidence showed a similar risk of IE after TAVR or SAVR.

The aim of this narrative review was to provide a detailed overview about IE in patients who underwent SAVR or TAVR, in order to assess the etiology and current treatments for IE aortic valve intervention and outline the outcome of these patients.

## 2. SAVR Endocarditis: Epidemiology, Pathogens, Medical Treatment

Prosthetic Valve Endocarditis (PVE) is one of the most dreadful complications after surgical aortic valve replacement (SAVR) [9].

The epidemiological outline of PVE after SAVR is challenging and it quite differs in relation to data from different international registries/studies (Table 1); in particular, the different works show great variability in the reported incidence rates, and this has an impact on proposing a definite value. Because SAVR still remains the most performed cardiac surgical intervention with more than 200,000 procedures per year worldwide, the risk for developing PVE is comparably higher [10,11,12].

The FinnValve Registry [13] enrolled more than 6400 patients who underwent TAVR or SAVR between 2008 and 2017. Among the 4333 patients who underwent bioprosthesis implantation via SAVR, the occurrence of PVE was about 2.9 per 1000 person-years during a mean follow-up period of 4.2 ± 2.6 years [13].

Luehr et al. [14] evaluated native valve endocarditis (NVE) vs. PVE after SAVR in a ten-year observational study (2005–2015); they recognized a 48.7% increase in PVE incidence (from 7.4 ± 3.9 to 11.4 ± 5.4 cases/year) within the last five years (2010–2015). According to patients’ characteristics, most of them were males (87.4% vs. 75.3%; *p* = 0.015) and older (67.9 ± 12.1 vs. 60.7 ± 14.7 years; *p* < 0.001) when compared to NVE patients; moreover, the PVE group showed a higher rate of single valve endocarditis (83.5% vs. 74.7%; *p* < 0.001) than NVE group [14].

A large retrospective French study analysed more than 100,000 patients undergoing isolated SAVR or TAVR for aortic stenosis (AS) from January 2010 to December 2018 [15]. Among the 60,253 patients who underwent isolated SAVR, PVE incidence was 1.40 (95% CI 1.34–1.46) events per 100 person-years with a lower global risk of developing IE after the procedure as compared with TAVR (RR 1.35, 95% CI 1.26–1.45) when considering unmatched populations [15]. Nonetheless, after adjusting the results by means of propensity score match analysis, the incidence rate of PVE was 1.71 (95% CI 1.58–1.85) events per 100 person-years in SAVR patients and there was no difference when compared to TAVR populations (RR 1.09, 95%CI 0.96–1.23) [15].

A sub-analysis from the randomized Placement of Aortic Transcatheter Valves (PARTNER)-I and -II trials and dedicated registries evaluated the occurrence of PVE in patients who underwent TAVR or SAVR procedures [16]. Among 8530 enrolled patients, there were 107 total cases of PVE: the incidence of PVE after SAVR was 4.10 per 1000 person-years, with no statistically significant difference with PVE after TAVR (*p* = 0.44) and a calculated incidence rate ratio (IRR) that is equal to 1.27, with SAVR being the reference point [16]. The authors also split data in relation to the timing of PVE occurrence after SAVR: most of the events (more than 60%) were during the period ranging from the 31st day to one year after the procedure; less than 10% of SAVR patients developed PVE within the first month after surgery, while the remaining patients suffered PVE after one year from the index surgical event [16]. In particular, the analysis from Kolte et al. [17] revealed an incidence of 2.5% (95% CI 2.3–2.9%) per person-year for the occurrence of early onset PVE after SAVR.

Indeed, after gathering the results from RCTs, Ando et al. [8] observed long-term incidence in PVE after SAVR that ranged from 0.6% after 2.0 years follow-up to 1.3% after 3.4-years follow-up. 

PVE is considered to be the worst complication after heart valve surgery, since it is still weighted with high early and late mortality, despite therapeutic and diagnostic improvements over time [19,20]. Luehr et al. [14] demonstrated overall in-hospital mortality for SAVR PVE equal to 22.3% (4.6% for elective cases and 17.5% for urgent/emergent cases), which increased until 25.2% during the follow-up period. Such percentages were influenced by the occurrence of post-operative complications, such as permanent renal failure (20.4%), sepsis and/or systemic inflammatory response syndrome (SIRS) (27.2%), low cardiac output syndrome (LCOS) (15.5%), and the need for ECLS/ECMO support (12.6%) [14].

Fauchier et al. [15] reported 32.78%/year all-cause mortality for PVE after SAVR; in particular, when analysing the timing after PVE diagnosis, the all-cause mortality was 14.81% after 30 days and 30.13% after one year. Similar results were obtained by Leontyev et al. [21]: among 313 patients undergoing redo SAVRs from December 1994 to April 2008, 48.6% was affected with PVE, showing a mean hospital mortality rate that is equal to 24.3%, which was increased in the case of clinical/post-procedural complication (complicated 30.9% vs. uncomplicated 12.7%; *p* = 0.01). Periannular abscess, for example, dramatically increased mortality (40.6% vs. 12.5%; *p* < 0.001) [21]. Finally, the mortality rate after surgical intervention for PVE still persists higher both within the first year (about 48%) and after ten years follow-up (about 69%) [21].

### 2.1. Pathogens in SAVR-IE

The identification of the causal infective agent of surgical PVE is a further challenging issue. Beyond the limitations deriving from IE related to fastidious microorganisms (i.e., HACEK bacteria) and/or intra-cellular bacteria—which can notably provoke the negative result of the analysis, most of patients suffering PVE underwent empirical antibacterial treatments, which are able to further promote difficulties in correctly identifying pathogens [22].

The direct culture of specimens from surgical biopsies may promote a better and more reliable identification of the outer microorganism.

Literature portrays different microbiological profiles for SAVR and TAVR PVE [13,14,16,17]. According to SAVR, data from registries and international trials provide insights about the causative agent (Table 2) [13,14,16,21].

A dedicated sub-analysis from PARTNER trial outlined that 58.3% of SAVR-PVE were caused by Staphylococcus, followed by Enterococcus (25%) and Streptococcus (8.3%) [16]. Indeed, the pathogen was not identified in approximatelt 8% of the cases [16].

The FinnValve Registry [13] revealed that Staphylococci were the most frequent cause of PVE after SAVR (41.5% of cases), with Coagulase-Negative (CoN) species being equal to 26.4%. Furthermore, Streptococci were responsible in 22.6% of cases, while Enterococci in 17%. A further seventeen percent were finally due to other causes (including blood culture negative IE) [13].

A retrospective study from Leontyev et al. [21] focused on causative agents also in relation to PVE timing: Staphylococcus species (spp), especially Aureus and CoNs, were mostly observed in both early (49%) and late (34%) PVE, as well as Enterococcus spp (21% vs. 18%), while Gram-negatives could only be found in a few cases (7%) of late PVE; Streptococci were more likely to be the cause for late PVE (16% vs. 8% for early PVE) [21].

This etiological distribution has been confirmed in a recent retrospective study from Luehr et al. [14], as these authors observed that Staphylococci (37.9%), Enterococci (15.5%), and Streptococci (12.6%) were the most common etiologic agents.

### 2.2. Medical Approach and Prognosis in SAVR-IE

The final management of PVE after SAVR needs a multidisciplinary approach by a dedicated “Endocarditis” team—in agreement with international guidelines—in order to individualize intervention in a tailored-suited manner [5]. Many aspects of the antimicrobial management are on empirical bases, given the lack of clinical trials testing medical treatments, especially for PVE caused by resistant pathogens.

The current guidelines do not significantly differentiate the medical managements of both NVE and PVE, except for PVE, due to Staphylococci, where the therapy should include rifampicin whenever indicated [5].

Bille [23] suggested a combination of three antibiotics (vancomycin or oxacillin, gentamicin and rifampicin) for staphylococcal PVE for at least six weeks. A case report from de Feiter et al. [24] reported the successful use of linezolid for the treatment of Staphylococcus epidermidis PVE after the failure of treatment with oxacillin, gentamicin, rifampicin, vancomycin, and fusidic acid regimens.

More recently, some authors focused on the prognostic assessment, in order to identify the high risk categories that may need more aggressive strategies [13,14,21].

Leontyev et al. [21] identified sepsis (odds ratio [OR]: 6.5), left ventricle ejection fraction (LVEF) less than 30% (OR: 5.8), concomitant coronary artery bypass grafting (CABG) (OR: 3.3), and aortic root abscess (OR: 2.7) as independent predictors of perioperative mortality for SAVR PVE, whereas sepsis (OR: 3.1) and unstable preoperative status (OR: 1.8) were shown to be predictors of long-term mortality. In this study, the patients with PVE showed a higher risk profile, as they were older, with more urgent/emergency cases and a higher incidence of preoperative neurologic dysfunction, thromboembolic events, renal failure, diabetes, and congestive cardiac failure. All of these conditions may explain the lower five-year survival rate reported [21]. Luehr et al. identified urgent surgery as an independent risk factor for in-hospital mortality (OR 6.461), while the identification of the causal pathogen was considered to be a protective condition for the positive outcome of the patients [14].

Indeed, Moriyama et al. [13] outlined the protective role of surgical intervention against the risk of mortality in patients with aortic PVE. Roughly, all of these results can be mainly attributed to the fast identification of the correct anti-microbial therapy—thus explaining the protective role of early identification of pathogens by means of preoperative blood cultures [14]—and the early indication to surgical intervention before patients’ decompensation [13].

Such findings were in line with Grubitzsch et al. [25], who stated that prompt diagnosis and subsequent treatment were fundamental in reducing morbidity, mortality, and, consequently, costs after PVE surgery.

## 3. TAVR Endocarditis: Epidemiology, Pathogens, Medical Treatment

The occurrence of IE on transcatheter-implanted prostheses is a rare complication, although the impact on prognosis is devastating [5].

The incidence and prevalence of IE after TAVR is difficult to determine, due to the recent introduction of the procedure in clinical management of aortic stenosis; indeed, data regarding incidence are quite uneven amongst the different studies (Table 3). Large cohort registries and observational studies provided a first glance of the impact of IE after the TAVR procedure [13,16,17,18,26,27,28].

A pooled cohort of all patients in PARTNER-I and PARTNER-II trials and registries observed a PVE incidence equal to 5.21 per 1000 person-years in patients who underwent TAVR, with most of them occurring during the first year after implantation (56.8% within one year vs. 43.2% after one year) [16]. The same results were reported by a large multicentre Italian registry, which enrolled 2572 consecutive patients who underwent TAVR, with no difference [26] in the incidence of PVE according to the type of transcatheter aortic prosthesis (i.e., balloon-expandable or self-expandable) [26]. Indeed, Stortecky et al. [27] showed a higher incidence in PVE after TAVR during the peri-procedural period with a 2.59 events per 100 person-years.

The FinnValve registry outlined an incidence of PVE that is equal to 2.4 per 1000 person-years among 2130 individuals who were treated with TAVR [13]. An incidence rate of early PVE equal to 1.7% was noted in a cohort of 29,306 patients collected by Kolte et al. [17].

Butt et al. [18] calculated a cumulative one-year risk of PVE equal to 2.3% in TAVR patients, with a cumulative five-year risk of IE that is equal to 5.8%. Similar results came from a retrospective analysis involving 1820 patients who underwent TAVR: the cumulative incidence rate of PVE was 3.02%, while most of them (74.5%) were within the first year after the procedure [28].

A recent meta-analysis from Wang et al. [29] reported an incidence rate ratio of 0.69 of IE after TAVR as compared to SAVR (*p* = 0.011), with the one-year post-TAVR incidence of IE being equal to 0.9%.

Data from the national TAVI registry SWENTRY (SWEdish traNscatheter cardiac intervention regisTRY), which is a sub-registry of SWEDEHEART (Swedish Websystem for Enhancement and Development of Evidence-based care in Heart disease Evaluated According to Recommended Therapies), found a 1.4% increased risk for PVE after TAVR within the first year, which lessened to 0.8% thereafter [30].

Finally, a comprehensive meta-analysis from Khan et al. [31] pointed out a mean incidence in PVE after TAVR of 3.25% (range interval: 0–14.3%).

IE in patients with valvular prostheses, surgical or transcatheter, is strictly associated with an increased burden of mortality during the follow-up [16]. The in-hospital mortality of PVE after TAVR is still high and above 60% than in patients who had an uncomplicated TAVR procedure [26,28,31,32]. Data from the SwissTAVI Registry reported a 6.55-fold higher risk for all-cause death in patients with PVE, with most of them occurring within 30-days after hospital admission (6.20-fold risk increase) [27]. The great impact on prognosis was mostly related to the time of PVE onset: peri-procedural PVE accounted for the majority of death (7.19-fold risk increase) when compared to delayed- or late-onset PVE [27].

Moreover, TAVR PVE was responsible of 4.03-fold risk increase in stroke, which reached higher values in late-onset IE after TAVR (11.92-fold risk increase) [27].

Indeed, the FinnValve Registry reported a cumulative increase in mortality rate related to TAVR PVE, ranging from 37.7% within 30-days after diagnosis to 52.5% one-year after [13]. Interestingly, the surgical approach to TAVR PVE seemed dramatically improving the in-hospital mortality rate of the patients by providing a 66% decrease in death rate [13].

Similar results were observed in a recent meta-analysis, which demonstrated a 37.8% rate of in-hospital mortality [29], mainly driven by heart failure during hospitalization, stroke during hospitalization, prior valve surgery, and Staphylococcus-associated PVE.

A systematic analysis from Khan et al. [31] outlined in-hospital mortality due to TAVR PVE that ranged from 11% to 47.2%, mortality rate at follow-up from 11% to 75%, and heart failure occurrence from 20% to 67.9% [31].

It is hard to define the final determinants that are able to predict the risk for TAVR PVE and the occurrence of negative outcomes. The bias in studies that tried to determine PVE predictors were mainly related to the highest burden of comorbidities of patients who underwent TAVR. However, gender and age can effectively impact the occurrence of TAVR PVE and possibly death [27,29]. Comorbidities, such as peripheral artery disease [29] and/or chronic kidney disease [18], revealed a two-fold increase in adverse outcomes in patients with PVE.

For sure, technical features that are related to the procedure may promote the occurrence of IE. Paravalvular aortic regurgitation [34], the need for implantable cardiac devices [34], heart failure history [17], use of non-hybrid surgical room [27], sepsis, cardiac arrest, and/or major bleeding during TAVR hospitalization [17] are further conditions that are able to favour the occurrence of TAVR PVE.

Indeed, the type of implanted prosthesis seemed not to affect the rate of IE occurrence: a meta-analysis from Tinica et al. [32] showed no difference in terms of time-interval between prosthesis implantation and IE occurrence between the two types of valves (i.e., self expandable or balloon-expandable). The same results were found by Summer et al. [16] in their analysis from the PARTNER trials: the occurrence of TAVR PVE is not linked to the type of valve, while other comorbidities may promote the infection of the device.

### Pathogens in TAVR-IE

Studies tried to report the most frequent microorganisms that are responsible for TAVR PVE (Table 4). The Italian multicentre study from Latib et al. [26] found that staphylococci and enterococci were commonly involved in TAVR PVE (about 50%), while negative cultures were reported in about 30% of cases. While staphylococci were mostly responsible for early onset IE, late IE were mainly related to staphylococci and enterococci [26]. The SwissTAVI Registry [27] confirmed these data: early, peri-procedural, and late onset IE were mostly related to infections from staphylococci and enterococci, although the authors observed the Viridans-group streptococci as able to determine the occurrence of valvular infection in late IE after TAVR. The Nationwide Readmissions Databases (NRD) reported that Staphylococci (30.4%), Streptococci (29.9%), and Enterococci (20.5%) were usually involved in TAVR PVE [17].

The FinnValve Registry pointed out that streptococci were the microorganisms mostly involved in TAVR PVE (46.7%), followed by staphylococci and enterococci (26.7% and 26.7%, respectively) [13]. These data were confirmed by the analysis of PARTNER trials: as compared to SAVR, patients with TAVR PVE were infected by streptococci (28.4% vs. 8.3%) [16].

Gathering the results from literature, Khan et al. [31] finally demonstrated that Enterococci (25.9%), Staphylococcus aureus (16.1%), and coagulase-negative Staphylococcus species (14.7%) were the causative microbiological agents that are involved in TAVR PVE.

The approach to TAVR PVE is challenging. Antibiotic prophylaxis was explored as a possible option for minimising the occurrence of IE after TAVR. Data from the SwissTAVI registry reported higher prevalence (92.6%) in antibiotic prophylaxis in patients who developed IE [27]. Indeed, such prophylaxis was ineffective: most of the patients (77.2%) were on 1st or 2nd generation cephalosporins, which are efficient on staphylococci and streptococci. Nevertheless, enterococci might not be neutralized by such a kind of drug, just as Gram negative agents. Therefore, the need for widening the spectrum of antibiotics is crucial in preventing PVE.

## 4. Instrumental Diagnosis

The role of imaging in identifying and diagnosing infective endocarditis (IE) in patients with prosthetic heart valve (PHV) is crucial. Beyond clinical signs, instrumental evaluation is able to confirm clinical hypothesis and point out the extent of lesions and the presence of local complications [35,36]. The European Society of Cardiology (ESC) guidelines indicated transoesophageal echocardiography (TOE, class I evidence B) as the first instrumental approach for diagnosing suspected IE in PHV [5]. Echocardiography can identify vegetations, abscess, leaflet dehiscence, fistulas, pseudoaneurysm, and/or new aortic regurgitation [37], thus allowing for a complete evaluation according to the ESC modified criteria for the diagnosis of infective endocarditis [5].

Moriyama et al. revealed vegetation and abscess as the most frequent complications occurring when echocardiographic evaluation was performed in patients with suspected IE after either transcatheter aortic valve replacement (TAVR) or surgical aortic valve replacement (SAVR) [13]. Indeed, the lower rate of complications until eight-year follow-up for both procedures (0.6% in TAVR and 0.9% in SAVR, respectively, after propensity score matching), which accounted for the small sample size, limits any further consideration [13]. Furthermore, the higher rate in moderate or severe aortic and/or paravalvular regurgitation—well known risk factors for IE—in patients who underwent TAVR as compared to SAVR can theoretically contrast previous data [38].

SAVR and TAVR patients with suspected IE can both have benefits from TOE evaluation, as this technique has become widespread, is relatively inexpensive, and requires no radiation. Nevertheless, this technique has some limitations [39], as the differentiation between infectious and non-infectious vegetation is not always possible and the effective reproducibility of the technique is also questionable. Habets et al. observed acceptable performances of TOE in IE patients with PHV: sensitivity 82% and specificity 95%, respectively, according to the identification of vegetations, while sensitivity 86% and specificity 98%, respectively, according to identification of periannular complications [40]. The authors did not calculate the performances of echocardiography in subgroups of patients, such as those undergoing TAVR/SAVR or biological/mechanical valve implantation.

Nevertheless, the performance of echocardiography in TAVR has rarely been investigated. Miranda et al. [41] found poor performances in both transthoracic echocardiography (TTE, diagnostic percentage: 18%) and TOE (diagnostic percentage: 47%), with slightly better results (62%) when evaluating patients with definite IE according to modified Duke criteria. Such results resembled those from 250 patients with IE after TAVR: echocardiography was able to detect vegetations in 67.6% of them, with size being one of the most important predictors for identification [33]. In particular, TOE can mostly identify vegetations on the leaflets of the biological valve and, to lesser extent, to the stent frame. According to the data from Regueiro et al. [33], self-expandable valves were more prone to developing vegetations at the stent frame, while the balloon-expandable valve develops IE on the leaflet valve [33]. An interesting work from Spartera et al. [42] outlined reduced sensibility for echocardiography in detecting IE after TAVR: although the detection of hemodynamic signs (mean aortic pressure gradient > 20 mmHg and/or worsening in aortic regurgitation grade) showed sensitivity that is equal to 75%, the identification of morpho-functional signs (i.e., reduced leaflet motion, mass, leaflet thicknening, de novo periprosthetic anechoic cavities/thickened area, and/or leaflet erosion) demonstrated a variation in sensitivity from 25% to 62.5%. Specificity ranges from 46.7% to 62.6% according to hemodynamic signs, being higher (till 100%) when considering morpho-functional signs [42]. The combination of hemodynamic and morpho-functional signs can improve the identification of IE in TAVR: ignoring leaflet motion, the combination of haemodynamics with each of other signs showed positive predictive values (PPV) that ranged from 94.4% to 100%, with negative predictive values (NPV) ranging from 45.5% to 100% [42]. Nevertheless, the combination of TTE and TOE might be the optimum in instrumental management of patients with IE after TAVR, above all for the correct identification of prosthetic vegetations [26].

In order to overcome the limitations from echocardiography (i.e., prosthetic strands, echoes from stents and scaffolds, etc.), it is fundamental to include a multimodality imaging for the assessment of patients with suspected IE after TAVR/SAVR [5,36]. The authors recently proposed intracardiac echocardiography (ICE) for improving diagnosis [43]. Nevertheless, the invasiveness of this technique, possible complications that are related to the performance of the examination, the need for expertise, and the cost for the performance and instrumentations reduce application of ICE in clinical practice. According to international guidelines, cardiac computed tomography (CT), magnetic resonance imaging (MRI), and nuclear imaging (radiolabelled white blood cell SPECT/CT and/or 18F-FDG PET/CT imaging) can non-invasively improve diagnosis [5]. Previous studies revealed good performance of cardiac CT as compared to TOE and three-dimensional TOE (3D-TOE), independent from type of surgery and valvular substitution [44,45,46]. Indeed, while cardiac CT seems to overcome diagnostic pitfalls in mechanical valves as compared to TOE, both of the techniques demonstrated comparable performances when applied in analysis of IE in bioprostheses [47].

The use of cardiac CT allows for obtaining high temporal and spatial resolution as well as three-dimensional images; nevertheless, valvular prosthesis can promote artifacts, patients undergo radiation exposure as well as contrast administration and potential harm kidney function, while higher and/or irregular heart rate can alter the acquisition of the images [48]. Beyond the detection of IEs and their features, cardiac CT scan allows for planning cardiac surgery interventions, as shown in the reciprocal relationships among valvular plane, coronary arteries, fibrous trigone, and aorta. Little data are about the application of CT within the specific field of SAVR and/or TAVR. Fangman et al. [49] considered twenty-seven consecutive patients with suspected aortic prosthetic valve IE after SAVR. ECG-gated CT demonstrated very good agreement with TOE when considering the identification of thickened wall (k = 0.83), while reducing for dehiscence (k = 0.75), abscess (k = 0.68), and vegetation (k = 0.55) [49]. CT demonstrated good agreement when compared to surgical findings (k = 0.66). Interestingly, the combination of ECG-gated CT and TOE very much improved the identification of valvular feature of IE on aortic valvular apparatus, reaching the highest agreement levels (k = 0.88) [49]. Therefore, ECG-gated CT can effectively improve the diagnosis of IE in aortic IE by integrating the information from TOE; once more, it can be useful for the evaluation of coronary arteries before surgical interventions [50]. Nevertheless, no data are available regarding the application of cardiac CT in patients with TAVR and suspected IE. Despite some case reports [51,52], dedicated randomized controlled trials are needed to implement information about the application and reproducibility of cardiac CT in TAVR with IE. The same considerations are for other novel techniques that are still under investigation when considering the specific fields of diagnosing IE in TAR/SAVR.

## 5. Surgical Treatment: Indications, Techniques/Prostesis, Outcome

### 5.1. Surgical Indication

There are no specific guidelines focusing on the management and treatment of prosthetic valve endocarditis. All of the indications that can be driven from 2014 AHA/ACC guidelines [53], 2015 ESC/EACTS guidelines [5], and 2016 AATS consensus guidelines [54] follow the principles indicated for intervention on native heart valves endocarditis. Several factors, such as patients’ characteristics, the presence of systemic or local complication, and type of microorganism, concur to define the surgical indication, but, at the same time, they may have a different impact on whether and when to propose a surgical procedure. The decision to undertake a high-risk and complex reoperation in patients with prosthetic valve endocarditis may be difficult, as a multispecialty evaluation within the “Endocarditis Team” or “Heart Valve Team” is recommended in these cases, not only for the diagnosis and the initial management (IIa) [5], but also for the definition of a surgical plan (IB) [53].

Antibiotic therapy represents one of the cornerstones of the treatment of patients with infective valve endocarditis, however, in most of the cases of PVE, the clinical and anatomical presentation warrants an early surgical operation in the active phase.

Early surgery is variably defined. According to the AHA/ACC guidelines, it refers to an operation that is performed “during initial hospitalization before completion of a full therapeutic course of antibiotic”. Because a six-week antibiotic therapy is recommended in the case of prosthetic aortic valve endocarditis, this definition includes a wide range of potential scenarios. ESC/EACTS guidelines provide a more detailed definition by considering early operation a procedure “on an emergency (within 24 h) or urgent (within a few days, <7 days) basis, irrespective of the duration of antibiotic treatment” and including surgical procedures that are postponed after one or two weeks of antibiotic treatment.

Surgery in the active phase is generally indicated to treat and avoid the progression of heart failure (i.e., valve dysfunction), to prevent embolism, in the case of uncontrolled infection (development of periannular complications, poor sepsis control) or in presence of microorganisms at low likelihood of being controlled by antimicrobial therapy (S. aureus, fungi). These conditions are common in prosthetic valve endocarditis. Prosthesis dehiscence is associated with severe aortic regurgitation in 40% of the cases while in 30% of the patients the presentation is complicated by heart failure [5,55]. Vegetations are described in up to 60–70% of the cases with evidence of systemic embolization in at least one-third of the patients and symptomatic cerebral embolism in approximately 20% of the cases [19]. Annular abscess and aorto-ventricular discontinuity are reported in 40% of the patients with a diagnosis of prosthetic aortic valve endocarditis and in up to 80% of the patients who ultimately underwent redo aortic surgery [21,56]. S. aureus and coagulase negative staphylococci are the causative microorganism in about 40% of the cases and, with fungal infection, they are involved in more than half of the cases of early prosthetic valve endocarditis.

Despite that a surgical option is commonly indicated in these patients, a redo procedure is performed in about 40–50% of the cases [20,57,58,59]. Alongside the coexistence of severe comorbidities and the presentation with life-threatening complications (i.e., intracranial haemorrhage, profound sepsis), patients’ refusal, cerebral embolism, and a perceived high surgical risk are the most common factors leading to a conservative management [60]. Failure to undertake surgery in patients with a potential indication for redo aortic valve replacement has been associated with a significantly worse early survival with a mortality of 40% in the first six weeks since the diagnosis [20,58,60]. A selection bias is expected in these retrospective observational studies, as patients precluded from a surgical option might have been in poor clinical condition. On this basis, patients who were denied a surgical procedure were usually older, with a longer history of chronic heart disease and severe comorbidities. However, when compared with patients who ultimately underwent an urgent redo operation, they presented less frequently with acute or worsening heart failure, mitral valve involvement, and prosthetic or periannular complications [20,58]. Not all of the patients who ultimately received a conservative management were inoperable. The decision to delay a surgical treatment, aiming at optimization of the clinical conditions while being supported by an initial positive response to lone medical therapy, might have exposed these patients to new embolic complications, the local evolution of the infective process, and worsening of heart failure and sepsis, thus leading to sudden death or a deeply deranged clinical status making an emergency surgical option worthless.

Limited evidence exists in the literature regarding the benefits of a surgical approach over a conservative treatment. The only available randomized clinical trial involving patients with infective endocarditis reported a better outcome for early surgery vs. medical treatment in patients with left-sided valve endocarditis, severe valve dysfunction, and large vegetations [61]. Similarly, a prospective, observational multicentric study found a significantly better survival in patients who had surgical valve replacement when compared to medical therapy, and this finding was also confirmed when stratified for the degree of patients’ illness [62]. A deeper knowledge of the outcomes and causes of death in patients who receive lone medical therapy could better underline the importance of maximizing the chance of providing a surgical option to patients with prosthetic valve endocarditis.

In the daily clinical practice, nowadays an early operation is generally advocated and widely accepted, as it is generally associated with a better survival [63,64,65]. This concept also applies to patients presenting with neurologic complications. Despite a great heterogeneity in reporting interval times (definition of early surgery) and the index event (cerebral complication or hospital admission), a surgical treatment in the first five to 14 days since the manifestation of the central embolic event, has not been associated with a higher risk of perioperative neurologic complications and mortality [66,67,68]. Therefore, a delayed operation >4 weeks is still recommended only in the case of intracranial haemorrhage [5].

### 5.2. Pathology and Surgical Treatment

The evolution and a wider availability of imaging tools leading to an early diagnosis and the general improvement in the treatment of sick patients with comorbidities have certainly enhanced the possibility of offering a successful surgical treatment in the case of complicated prosthetic aortic valve endocarditis. However, despite these advances, aortic PVE is still associated with substantial early morbidity and mortality.

Local extension of the infective process is commonly reported in aortic PVE. A periannular abscess, a region of necrosis with purulent material without luminal communication, is described in up to 80% of the cases [21,56,69,70,71]. A further progression of the infective process may cause tissue liquefaction with perforation and fistulization in other cardiovascular structures, or the development of a false aneurysm, a contained rupture that is surrounded by the heart and mediastinal vessels [70,72]. The separation between the aorta and left ventricle of more than one-third of the annular circumference defines the presence of aorto-ventricular discontinuity [73]. This condition is associated with a wide tissue destruction and poor clinical conditions, with patients generally experiencing severe heart failure, uncontrolled sepsis, and mechanical complications, such as chest pain or syncope, due to systolic collapse of the aortic root and/or ascending aorta [72,73,74]. A pseudoaneurysm involving the intervalvular fibrosa and both aortic and mitral valves is a further well-defined complication that is characterised by a pulsatile cavity (systolic expansion and diastolic collapse) in the mitral-aortic junction in communication with LVOT. Finally, subannular extension of the infective process can also lead to LVOT disruption, ventricular septal defect, and intracardiac shunts [69,75,76].

The primary objectives of surgical treatment are radical debridement and the removal of infected tissues, the reconstruction of cardiac and aortic morphology, and the restoration of the aortic valve function.

The radical removal of infected and frail tissue and reconstruction of the aortic annulus allow for a secure implantation of an aortic prosthesis and the control of the infection with a freedom from recurrent endocarditis and reoperation of 86% and 70%, respectively, at 15-year follow-up [77,78].

In the case of extensive involvement of the aortic root and aorto-ventricular discontinuity, a full root replacement is invariably required, especially when the abscess is located at the left cusp area, the interventricular septum or cause a circumferential disruption of the aortic annulus [56,79,80]. These lesions are more common in patients with a previous history of endocarditis or an uncontrolled infection and often require an urgent reoperation. Redo root operations for infective endocarditis present several technical challenges. Chest re-entry can be difficult in the presence of aortic false aneurysm or strong adhesions involving the mediastinal structures. The preparation and reconstruction of the neo aortic annulus can be complicated by damage of the pulmonary and coronary arteries. Particularly, myocardial protection is crucial and it includes the avoidance of distension of the left ventricle, adequate cardioplegic arrest, and successful restoration of coronary flow. Failure in myocardial protection represents one of the most important risk factors for early mortality [79]. Redo surgery is burdened by a postoperative mortality up to 20%. However, when considering these complex medical and surgical scenarios, it represents the only valuable option of survival in these sick patients and provides a satisfactory mid-term survival and freedom from infection relapse [56,72,74,80,81,82].

The involvement of the intervalvular fibrosa requires the reconstruction of the cardiac fibrous skeleton and restoration of aortic and mitral valves function. Techniques and principles for dealing with these challenging cases were posed in the early 1990s with the evidence that a double valve replacement with an extensive pericardial patch repair could be effective in the treatment of complicated heart valves infection [83,84,85]. Recent experiences have confirmed the efficacy of these procedures. Perioperative mortality is still high (around 20%), but mid-term outcomes are satisfactory, especially when considering the characteristics of these patients and the magnitude of the infective process, with a survival of 60% and freedom from reoperation and recurrent infective endocarditis of 80%, at 10-year follow-up [86,87,88]. Mitral repair has been performed instead of mitral valve replacement in the case of limited involvement of the mitral leaflets and it has been associated with a lower incidence of early postoperative complications and a better survival in this cohort of patients [88].

The choice of type of prosthesis for valve replacement in infective endocarditis has been a matter of debate for decades. Important tissue loss and necrosis and the need for extensive resection and debridement have been generally treated with the use of biologic materials.

The use of homografts has been primarily favoured, owing to a presumed high resistance to infection; however, no evidence exists regarding a significant advantage in preventing a recurrence of infective endocarditis [89]. No difference in survival and reoperations were reported between patients, who received an allograft and patients who underwent mechanical aortic valve replacement for infective endocarditis [90,91]. However, these data derived from populations with predominantly native aortic valve endocarditis and with a higher rate of periannular abscess and more severe disease in the homograft groups. The risk of a reoperation for progressive deterioration of an aortic allograft becomes significant in the second decade since its implantation and, in the case of diffuse calcific degeneration, may expose the patients to a high-risk and difficult procedure [92,93]. Regardless of these potential problems in the long run, the use of an allograft should be considered in the case of complex root pathology and extensive tissue disruption, as its pliable tissue can ease the anatomical repair and the attached anterior mitral leaflet can be used in reconstruction of the fibrous skeleton of the heart.

The mechanical and biological prostheses have led to similar results in terms of survival, persistent infection, and relapse of infective endocarditis [94]. Some evidences reported a survival advantage for mechanical valves; however, these findings were driven from unmatched populations, with the patients undergoing biological aortic valve replacement at higher risk and older [64,95,96]. No special recommendations exist in aortic PVE for the type of valve choice; patients’ preference and the baseline clinical and anatomical conditions should guide the decision balancing the expectation of possible problems in the mid- and long-terms (i.e., difficult re-redo for biological valve degeneration) and the early management (i.e., the avoidance of postoperative anticoagulation in patients with a recent embolic stroke).

In experienced hands, the Ross procedure may be used in non-elderly adults. Recent reports have shown that an autograft aortic valve replacement can be safely performed both in patients with infection that is limited to the aortic cusps and in cases of aortic PVE complicated by periannular abscesses. In these well-selected populations of patients, survival and freedom from recurrent endocarditis was 85% and 89%, respectively, at 10-year follow-up. Although the Ross procedure could offer some benefits in the long run, in the context of uncontrolled sepsis, worsening heart failure, this kind of procedure is not indicated, as it is more appropriate to minimise the surgical insult in order to increase to chance of recovery and early survival [97,98].

Finally, in extreme cases, cardiac transplantation may be considered if infection persists, despite multiple surgical attempts or if a satisfactory anatomical and functional restoration appears to be impossible [99].

### 5.3. Treatment of IE after TAVR: Emerging Evidence

The outcomes of patients with infected TAVR prosthesis have been reported invariably poor with a survival rate of 30% after one year/two years since the diagnosis [33,100]. Despite this dismal natural history, almost 90% of the patients with TAVI endocarditis did not receive any further interventional treatment. Few cases of successful repeat TAVI procedure after an adequate antibiotic therapy have been reported, and limited evidence are available regarding patients who had a surgical operation [100], despite the vast majority presenting with clinical and anatomical features supporting a surgical indication [100,101,102].

Two previous studies found no difference in terms of early outcome between patients who had conservative and surgical management for TAVI IE [102,103]. Several limitations apply to these findings: the populations involved were relatively small, there was a high heterogeneity between the two groups of patients in terms of preoperative clinical status and the presence of anatomical complications, and, finally, the follow-up time was limited to three and six months. A preliminary analysis of STS database found a postoperative mortality of 29% in 138 patients who underwent TAVI retrieval for infective endocarditis and reported that a concomitant procedure for aortic or mitral repair was required in more than two-thirds of the cases [104]. These results are in line with the experiences in redo operation for surgical aortic valve prosthesis infection and they account for the inclusion of elderly patients with multiple comorbidities and, as per the initial practice in trans-catheter aortic valve procedure, often deemed to be inoperable in an elective setting. Furthermore, periannular extension of the infective process is common in TAVI endocarditis, as it was described in more than one-third of the cases [100,102]. A peculiar involvement of the mitral valve has been reported in approximately 30% of the patients with the presence of vegetations, perforation of the anterior mitral valve leaflets, or the development of pseudoaneurysm of the intervalvular fibrosa. A complex surgical repair is invariably required in these cases, and it may increase the risk of the surgical treatment and the time of recovery. Other technical issues are associated with the type of the implanted TAVI prosthesis. The explantation of both self-expandable and balloon-expandable degenerated valves can be performed with a cautious mobilization of the prostheses from the aortic annulus and the aortic root [105,106]. However, in the case of infective endocarditis, especially for self-expandable prostheses, a high rate of aortic root and ascending aorta replacement has been described, due to aortic wall disruption, the presence of abscess, or development of aortic false aneurysm [33,100,104,107].

No special guidelines or recommendations have so far addressed the management of TAVI IE. These initial findings showed that the infection of a transcatheter prosthesis shares many features with surgical aortic prosthesis endocarditis. The surgical results are still sparse and based on high-risk populations or elderly patients; however, they suggest that the consolidated strategies that were developed for surgical aortic PVE can be applied in this setting and could be the only appropriate and successful treatment for transcatheter aortic valve infection.

## 6. Final Considerations

Infective endocarditis on prosthetic valves still remain the most worrisome complication after both the SAVR and TAVR procedures. Published studies do not reveal whether either technique is riskier in determining infective endocarditis. Indeed, IE after TAVR or SAVR can dramatically affect the survival rate of patients, even despite adequate antibiotic therapies and/or further surgical, re-do interventions. Adequacy in antibiotic prophylaxis before the two interventions, correct selection of the candidates, fast diagnosis, and prompt interventions might be considered to be the cornerstone in preventing the negative consequences of IE.

## Figures and Tables

**Table 1:** Characteristics of the main studies dealing with infective endocarditis in surgical aortic valve replacement (SAVR).

Reference	Study Design	SAVR Population (n)	Period	Epidemiological Data	Outcomes	Associated Conditions
Moriyama et al. (2019) [13]	Retrospective (FinnValve registry)	4333	2008–2017	Incidence IE: 2.9/1000 person-yrs	In-hospital death: 32.1%	-Male gender (HR 1.73, 95% CI: 1.04–2.89) -Deep sternal wound infection/vascular access-site infection (HR 5.45, 95% CI: 2.24–13.2) -Hospital death (HR 0.34, 95% CI: 0.21–0.61)
Luehr et al. (2019) [14]	Retrospective, observational	103	2005–2015	IE incidence 2005–2010: 7.4 ± 3.9 cases/yrs IE incidence 2011–2015: 11.4 ± 5.4 cases/yrs	Overall mortality: 47.6% In-hospital mortality: 22.3% Follow-up mortality: 25.2%	Mortality risk factors: Urgent surgery; Mitral regurgitation II; Previous cardiac operation with homograft; LVEF < 40%
Fauchier et al. (2020) [15]	Retrospective, propensity matched (French registry)	60,253 (propensity: 16,291)	2010–2018	UNMATCHED Incidence IE: 1.40/100 person-yrs MATCHED-PROPENSITY Incidence IE: 1.71/100 person-yrs	MATCHED-PROPENSITY All-cause death 32.8%	Male gender, Charlson comorbidity index, frailty index, obesity, alcohol abuse and presence cardiac implantable electronic device
Summers et al. (2019) [16]	Cohort study PARTNER RCTs and registries	1257	2007–2016	Incidence IE: 4.10/1000 person-yrs	All-cause mortality risk: HR 12.03, 95% CI, 5.15-23.51	Cirrhosis Significant pulmonary disease CKD
Kolte et al. (2018) [17]	Retrospective, propensity matched (U.S. Nationwide Readmissions Databases)	66,077 (propensity: 6942)	2013–2014	UNMATCHED Incidence IE: 2.5/100 person-yrs MATCHED Incidence IE: 1.9/100 person-yrs	In-hospital mortality: 15.6%	Younger age History heart failure Need permanent PM Cardiac arrest Major bleeding Sepsis
Butt et al. (2019) [18]	Nationwide observational cohort study	3777	2008–2016	Incidence IE: 1.2/100 person-yrs 5-year IE risk: 5.1% (95% CI: 4.4% to 6.0%),	In-hospital mortality: 14.0% 1-year mortality 23.1%	Male sex and diabetes

Abbreviations: CI: confidential interval; HR: hazard ratio; IE: Infective endocarditis; PARTNER: Placement of Aortic Transcatheter Valves trial; RCT: Randomized Controlled Trial; SAVR: surgical aortic valve replacement; U.S.: United States; Yrs: years.

**Table 2:** Characteristics of the main studies dealing with infective endocarditis in surgical aortic valve replacement (SAVR).

Reference	Staphylococcus Aureus	Coagulase Positive Staphylococcus	Coagulase Negative Staphylococcus	Enterococcus	Streptococcus	Others
Moriyama et al. (2019) [13]	/	15.1%	26.4%	17.0%	42.6%	18.9%
Fauchier et al. (2020) [15]	/	17.3%	15.5%	21.2%	24.3%	8.6%
Summers et al. (2019) [16]	58.3%	/	/	/	8.3%	/
Luehr et al. (2019) [14]	32.2%	/	/	14.2%	21.5%	/

**Table 3:** Characteristics of the main studies dealing with infective endocarditis in transcatheter aortic valve replacement (TAVR).

Reference	Study Design	Population (n)	Period	Epidemiological Data	Outcomes	Associated Conditions
Moriyama et al. (2019) [13]	Retrospective (FinnValve registry)	2.130	2008–2017	Incidence IE: 3.4/1000 person-yrs	In-hospital deah: 20.0%	-Male gender (HR 1.73, 95% CI: 1.04–2.89) -Deep sternal wound infection/vascular access-site infection (HR 5.45, 95% CI: 2.24–13.2)
Regueiro et al. (2016) [33]	Retrospective (Infectious Endocarditis after TAVR International Registry)	20,006	2005–2015	Incidence IE 1.1% per person-yrs Incidence early IE 0.9% per person-yrs	-Surgery during index hospitalization: 14.8%, 95% CI, 10.4–19.2% -Surgical transcatheter valve explantation: 10.8%, 95% CI, 6.9–14.6% -TAVR valve-in-valve: 1.2%, 95% CI, 0–2.5% -Antibiotic therapy alone: 82.0%, 95% CI, 77.2–86.8% In-hospital death: 36%, 95%CI, 30.0–41.9%. 2-year mortality: 66.7%, 95% CI, 59.0–74.2%	-Male gender (HR, 1.69; 95% CI, 1.13–2.52) -Age (HR, 0.97; 95% CI, 0.94–0.99) -Diabetes (HR, 1.52; 95% CI, 1.02–2.29) -residual moderate/severe aortic regurgitation (HR, 2.05; 95% CI, 1.28–3.28)
Latib et al. (2014) [26]	Retrospective on multicenter registry	2572	2008–2013	Incidence IE: 1.13% [95% CI: 0.76% to 1.62%] According to IE onset: -Early (<60 days): 28% -Intermediate (60–365 days): 52% -late (>365 days): 20%	Overall mortality: 62% In-hospital mortality: 45% Follow-up mortality: 17%	N/A
Fauchier et al. (2020) [15]	Retrospective, propensity matched (French registry)	47,553 (propensity: 16,291)	2010–2018	UNMATCHED Incidence IE TAVR: 1.89/100 person-yrs MATCHED-PROPENSITY Incidence IE TAVR: 1.86/100 person-yrs	MATCHED-PROPENSITY All-cause death: 43.0%	Male sex, Charlson comorbidity index, frailty index, AF and anaemia
Summers et al. (2019) [16]	Cohort study of PARTNER RCTs and registries	7273	2007–2016	Incidence IE: 5.21/1000 person-yrs	All-cause mortality risk: HR 4.09, 95% CI, 3.09–5.41	Cirrhosis; significant pulmonary disease; CKD
Kolte et al. (2018) [17]	Retrospective, propensity matched (U.S. Nationwide Readmissions Databases)	29,306 (propensity: 6942)	2013–2014	UNMATCHED -Incidence IE: 1.7/100 person-yrs MATCHED -Incidence: 1.7/100 person-yrs	In-hospital mortality: 15.6%	Younger ag History heart failure Need permanent PM Cardiac arrest Major bleeding Sepsis
Butt et al. (2019) [18]	Nationwide observational cohort study	2632	2008–2016	IncidenceIE: 1.6/100 person-yrs 5-year IE risk: 5.8% [95% CI: 4.7% to 7.0%]	In-hospital mortality: 20.9% 1-year mortality: 40.0%	Male sex and CKD
Stortecky et al. (2020) [27]	Retrospective (SwissTAVI Registry)	7203	2011–2018	INCIDENCE -Peri-procedural (<100 days): 2.59/100 person-yrs -Delayed-early (100–365 days): 0.71/100 person-yrs -Late (>365 days): 0.40/100 person-yrs Overall 5-years incidence: 1.0/100 person-yrs	All-cause mortality risk: -Overall: HR: 6.55 (95% CI: 4.44–9.67) -Peri-procedural IE: HR: 7.19 (95% CI: 3.69–14.03) -Delayed IE: HR: 5.05 (95% CI: 2.10–12.16) -Late IE: HR: 7.34 (95% CI: 4.13–13.05) Stroke risk: -Overall: HR: 4.03 (95% CI: 1.54–10.52) -Peri-procedural IE: HR: 1.28 (95% CI: 0.23–7.24) -Delayed IE: 0 -Late IE: HR: 11.92 (95% CI: 2.76–51.53)	-Younger age -Male gender -Lack predilatation balloon aortic valvuloplasty before valve implantation -Treatment in cath-lab as opposed to hybrid
Mangner et al. (2016) [28]	Retrospective	1820	2006–2014	Cumulative incidence: 1.82/100 patient-yrs	In-hospital mortality:63.6% 1-year mortality: 74.5%	-Chronic hemodialysis -PAD
Bjursten et al. (2019) [30]	Retrospective (TAVI registry SWENTRY)	4336	01/2018–06/2018	Incidence 1 year: 1.42% (1.03–1.80%) 1–5 yrs: 0.80% (0.60–1.06%) 5–10 yrs: 0.52% (0.20–1.32%)	1-year survival: 58% 5-year survival was 29%	Body surface area; eGFR < 30 mL/min/1.73 m^2^; Critical pre-operative state; mean pre-procedural valve gradient; Amount contrast dye; Transapical access; A.F.

Abbreviations: AF: Atrial fibrillation; CI: confidential interval; CKD: history chronic kidney disease; eGFR: estimated glomerular filtration rate; HR: hazard ratio; IE: Infective endocarditis; N/A: not applicable; PAD: peripheral artery diseases; PARTNER: Placement of Aortic Transcatheter Valves trial; PM: pacemaker; RCT: Randomized Controlled Trial; SWENTRY: SWEdish traNscatheter cardiac intervention registry; TAVR: transcatheter aortic valve implantation; TAVR: transcatheter aortic valve replacement; U.S.: United States; Yrs: years.

**Table 4:** Characteristics of the main studies dealing with infective endocarditis in transcatheter aortic valve replacement (TAVR).

Reference	*Staphylococcus aureus*	Coagulase Positive *Staphylococcus*	Coagulase Negative *Staphylococcus*	*Enterococcus*	*Streptococcus*	Others
Moriyama et al. (2019) [13]	/	20%	6.8%	26.7%	46.7%	0%
Regueiro et al. (2016) [33]	23.8%	/	16.8%	24.6%	/	/
Latib et al. (2014) [26]						
-Early-onset group	50%	/	50%	/	/	/
-Intermediate-onset group	/	20%	/	20%	20%	/
-Late-onset group	/	33%	/	33%	/	/
Fauchier et al. (2020) [15]	/	15.8%	13.2%	22.7%	29%	7.1%
Summers et al. (2019) [16]	28.4%	/	/	/	28.4%	/
Kolte et al. (2018) [17]	30.4%	/	/	20.5%	29.9%	11.1%
Stortecky et al. (2020) [27]	21.5%	/	/	26.2%	28.9%	/
Mangner et al. (2016) [28]	/	38.2%	9.1%	/	3.6%	18.2%
Bjursten et al. (2019) [30]	22.3%	34%	6.8%	20.4%	/	16.6%
